# Joint Attention and Play Development in Toddlers with Severe Visual Impairment: A Comparison with Sighted Peers

**DOI:** 10.3390/children13060804

**Published:** 2026-06-11

**Authors:** Hale Cotuk Yucel, Selda Ozdemir

**Affiliations:** 1 Department of Special Education, Nevsehir Haci Bektas Veli University, Nevsehir 50300, Türkiye; 2 Department of Special Education, Hacettepe University, Ankara 06800, Türkiye; seldaozdemir@hacettepe.edu.tr

**Keywords:** severe visual impairment, joint attention, play development, early childhood, developmental differences

## Abstract

**Highlights:**

**What are the main findings?**

- Toddlers with severe visual impairment demonstrated significantly lower joint attention skills and differences in play development compared with sighted peers matched for language developmental age.
- Significant associations were identified between joint attention and play skills in both groups, although these relationships were stronger and more advanced in sighted children.

**What are the implications of the main findings?**

- The findings highlight the importance of early assessment and intervention targeting joint attention and play skills in toddlers with severe visual impairment.
- Multisensory and play-based intervention approaches may support early social communication and developmental participation in children with severe visual impairment.

**Abstract:**

**Background:** Joint attention is a fundamental skill supporting early social communication and cognitive development. However, its development in children with severe visual impairment and its relationship with early play behaviors remain insufficiently understood. This study aimed to compare joint attention skills and play behaviors in toddlers with severe visual impairment and sighted peers, and to examine the relationship between these two developmental domains. **Methods:** The sample included 17 toddlers with severe visual impairment (no light perception) and 20 sighted toddlers aged 16–36 months. Groups were matched based on language developmental age scores. Joint attention skills were assessed using structured observational categories adapted to visual status, while play behaviors were evaluated with the Developmental Play Assessment. Data were collected through semi-structured play and interaction sessions in familiar environments to support natural behavior. Analyses were conducted using non-parametric tests, including the Mann–Whitney U test and Spearman correlation analysis. **Results:** Toddlers with severe visual impairment scored significantly lower than sighted peers across all joint attention categories. In play development, these children showed higher levels of indiscriminate actions, whereas sighted peers demonstrated more advanced play behaviors, including combinational and symbolic play. Significant associations were found between joint attention and play skills in both groups; however, these relationships were stronger in sighted children, particularly for higher-level play forms. **Conclusions:** These findings suggest distinct developmental trajectories and underline the importance of early intervention targeting these foundational skills in early childhood development and highlight important implications for early intervention practices.

## 1. Introduction

Early emerging joint attention skills are defined as the ability of two individuals to share a common focus on the same object or event [1]. Joint attention is typically conceptualized in two forms: initiating joint attention and responding to joint attention [2,3,4]. In initiating joint attention, infants direct others’ attention using gestures and eye contact, whereas in responding to joint attention, they follow others’ gaze and gestures to share a joint focus [5].

In typically developing infants, joint attention emerges between approximately 12–15 months of age [6], with responding to joint attention preceding initiating behaviors [7]. During this period, infants use gaze following, pointing, and head turning to coordinate attention, while relying on adult cues to interpret shared referents [8]. By 10 months, gaze-following becomes more consistent [9], although its effectiveness depends on gesture clarity and target location [10]. Visual cues, including eye gaze and facial expressions, play a central role in attention coordination [11,12].

By the end of the first year, infants begin to include adults in object-centered activities, imitate their interactions with objects, and use social referencing to interpret unfamiliar situations [13,14]. As interactions become more complex, they expand to include shared attention and joint action, supporting an understanding that objects and events can be shared between individuals [15]. Infants also begin to understand others’ intentions and increasingly engage in reciprocal and imitative interactions, contributing to shared meaning and early social development [16,17,18].

Visual information plays a critical role in joint attention by enabling infants to detect gaze direction and identify others’ attentional focus [11,19]. Gaze is a fundamental component of social interaction [20], and sensitivity to eye contact supports learning from others’ attention [21]. While younger infants primarily focus on objects, older infants begin to recognize both objects and others’ attentional states, marking the emergence of joint attention and early self-awareness [14].

However, the development of joint attention may follow a different trajectory in children with severe visual impairment due to reduced access to visual cues. The absence of behaviors such as gaze and pointing may affect the ability to coordinate attention and interpret others’ focus. Children with visual impairments tend to use gestures differently, showing more limited use of visually acquired deictic gestures [22], and may display less explicit responses in social interactions, along with limitations in joint attention and social referencing [23]. Therefore, they may experience early difficulties in establishing and maintaining joint attention, posing risks for later social and communicative development [24,25]. Reduced visual input may also affect broader socio-cognitive processes, including joint attention, emotion recognition, and observational learning [26,27,28]. Although children with visual impairments may possess adequate movement skills, limited access to visual information can reduce motivation for environmental exploration and contribute to delays in motor development, resulting in fewer opportunities for social interactions and learning [29,30,31]. For example, reaching behaviors may emerge later and rely more on cognitive processes than visual guidance [23,32].

The literature presents different perspectives on the development of joint attention in children with visual impairments. Some studies suggest delays due to limited visual inputs [33,34,35,36], whereas others indicate that joint attention may emerge within a similar developmental timeframe but through qualitatively different behaviors [37,38]. For example, children with visual impairments may respond to joint attention bids through alternative behaviors such as remaining still, leaning toward objects, or producing rhythmic movements [39].

Joint attention and play development are considered closely related developmental processes because both involve shared interaction, imitation, social participation, and the coordination of attention toward objects and people [40,41]. Through joint attention experiences, children learn to observe others’ actions, engage in reciprocal social exchanges, and construct shared meanings within play contexts [14,41,42]. These early social interactions contribute to the emergence of symbolic and representational play behaviors by supporting children’s ability to imitate actions, use objects symbolically, and participate in pretend play activities [41,42]. In typically developing children, opportunities to observe, imitate, and share attention during everyday interactions may therefore support the development of increasingly complex symbolic play behaviors [43]. In addition to supporting shared engagement, joint attention provides opportunities for children to observe and imitate the actions of others within social interactions [14]. Through these experiences, children gradually develop symbolic and representational play behaviors, including the ability to use objects symbolically and engage in pretend play activities [42]. Accordingly, imitation processes are considered important developmental foundations for symbolic play and early social-cognitive development [14].

Learning and development occur within social contexts, and play provides a central setting for these interactions [44,45]. Through play, children develop social and communicative skills, including shared attention and interaction [46]. In typically developing children, play is closely linked to joint attention, as children use shared focus and gestures to coordinate interactions and construct meaning [47]. In contrast, children with visual impairments may experience difficulties in play-based interactions. They may show limitations in sharing attention and experiences with others and exhibit differences in play behaviors compared to sighted peers [24,48,49,50,51,52,53,54,55]. These children tend to engage less in symbolic play, prefer tactile and auditory materials, and display more solitary play behaviors with a more limited play repertoire [48,50,53,54]. Accordingly, tactile and auditory experiences may become increasingly important for supporting social engagement, object exploration, and play participation in children with severe visual impairment [34,54].

Play development progresses from simple exploratory actions to more complex symbolic forms [56,57]. During the earliest stages of development, children often engage in indiscriminate exploratory behaviors such as mouthing, throwing, or manipulating objects in similar ways [54]. Over time, children begin to use objects according to their functional properties and gradually develop representational and symbolic play behaviors involving pretend actions, imitation, and shared social experiences [42,57]. Symbolic play is closely associated with social-cognitive skills such as imitation and joint attention [42,58]. Therefore, limitations in joint attention may directly influence the development and complexity of play. These differences are significant, as difficulties in play and peer interaction may lead to reduced social participation and potential social challenges, which underscores the importance of examining the relationship between joint attention and play [59,60]. Evaluating play alongside joint attention provides important insights into developmental profiles and supports the identification of early intervention targets [61,62]. Joint attention is also central to early communication, behavior regulation, social interaction, and the sharing of experiences [63,64]. It contributes to both language and social-cognitive development, while limitations in joint attention may impact broader developmental outcomes [65,66,67]. Assessing joint attention alongside play and communication skills therefore provides valuable information about children’s developmental functioning [68,69]. However, findings regarding children with visual impairments remain inconsistent, partly due to methodological problems such as difficulties in matching comparison groups on developmental variables [70,71]. Addressing research quality limitations is essential for a clearer understanding of developmental processes in young children with visual impairments.

Considering this, the present study aimed to evaluate joint attention and play skills in toddlers with severe visual impairments and to compare these skills with those of sighted peers matched for language developmental age. Additionally, the study examined the relationships between joint attention and play skills in both groups. By addressing these aims, the study seeks to contribute to a more comprehensive understanding of developmental profiles in children with severe visual impairment and to inform early intervention practices.

## 2. Materials and Methods

This study employed a quantitative cross-sectional comparative research design to examine joint attention and play skills in toddlers with severe visual impairment and sighted peers matched for language developmental age.

### 2.1. Participants

Visual impairment is commonly classified into two categories: severe visual impairment (blindness) and low vision [72]. Severe visual impairment refers to the absence of functional vision, including the lack of light perception [73]. Participants with severe visual impairment were recruited through special education and rehabilitation centers providing services for children with visual impairments. Children with additional diagnosed neurological, hearing, or developmental disabilities were not included in the study. The study included 17 toddlers with severe visual impairment who had no light perception and 20 sighted toddlers aged between 16 and 36 months. The groups were matched based on language developmental age to ensure comparability across developmental levels.

The demographic characteristics of the participants are presented in Table 1.

### 2.2. Measures

Children’s language development was assessed using the Turkish Communicative Development Inventory (TIGE) [74], adapted from the MacArthur–Bates Communicative Development Inventories. TIGE consists of two forms covering the age ranges of 8–16 months and 16–36 months. In the present study, the TIGE-II form (16–36 months) was used. The instrument is a standardized caregiver-report measure with established validity and reliability, as reported in the Turkish adaptation study [74]. TIGE-II data were obtained through structured caregiver interviews conducted by the researcher with the primary caregivers during the first assessment session. The TIGE provides information on multiple aspects of early language development, including vocabulary size, word use, morphological structures, multi-word combinations, grammatical markers, and sentence complexity. Based on the scores obtained, language developmental ages were calculated and used to match the groups.

Joint attention behaviors were assessed using the observational categories developed by Bigelow [33] for children with visual impairments. These categories are organized into three levels: Preliminary Behaviors, Liberally Construed Joint Attention Behaviors, and Conservatively Construed Joint Attention Behaviors. Preliminary behaviors reflect early interactions without clearly established shared attention, whereas liberally and conservatively construed behaviors represent increasingly coordinated and reciprocal forms of joint engagement. The materials used in these tasks were adapted from Ross [35] and included developmentally appropriate objects designed to elicit joint attention behaviors. For children with severe visual impairment, materials incorporated salient tactile and auditory features. To provide a more comprehensive evaluation, additional categories—Initiating Joint Attention (requesting) and Responding to Joint Attention—were incorporated based on Özdemir [75]. To enable comparable assessment across groups, orientation behaviors were operationalized according to the sensory characteristics of each group. For children with severe visual impairment, responses were coded based on orienting behaviors toward auditory or tactile stimuli (e.g., turning the head or body toward a sound source), whereas for sighted children, responses were coded based on visual orientation (e.g., looking toward a target). Each observational category was assessed across three trials. Items assessing initiating and responding to joint attention were presented throughout the observation rather than consecutively, with intervals of approximately 2–3 min between tasks. The original joint attention categories proposed by Bigelow [33] were retained without modification. Adaptations were limited to the materials and observational indicators used to ensure comparable assessment across groups while maintaining the original conceptual framework. An observation form was used to record children’s responses to joint attention tasks, with each response scored as 1 (observed) or 0 (not observed). Behaviors elicited through maternal prompts were excluded from coding. Only behaviors occurring in response to the structured joint attention tasks and social attentional cues presented during the interaction were coded as joint attention behaviors.

Play skills were evaluated using the Developmental Play Assessment [41], which examines play development across a hierarchical sequence of categories reflecting increasing levels of complexity. The assessment includes the following categories: Indiscriminate actions, Discriminative actions, Takes apart combinations, Presentation combinations, General combinations, Pretend self, Specific combinations, Child as agent, Specific combinations, Single-scheme sequences, Substitutions, Doll as agent, Multischeme sequences, Sociodramatic play, and Thematic fantasy play. Play behaviors were coded according to these categories, with higher-level play reflecting greater representational complexity. The materials used in the play assessment were developed based on the toy sets described by Lifter [56]. Two standardized toy sets were prepared to elicit a range of play behaviors. Set 1 included a doll, nesting cups, spoon, fork, blanket, mirror, comb, toy car, and a transparent container with cube and cylindrical blocks. Set 2 included animal figures (e.g., dog, sheep, cow, donkey), family figures (mother, father, child), a segmented toy train, a teapot, cup, plate, an animal-shaped puzzle, and a closed container with screws. All materials were selected to support exploratory, functional, and symbolic play and were used consistently across participants. For children with severe visual impairment, materials included salient tactile and auditory features. Each toy set was presented for approximately 10 min, resulting in a total play observation period of approximately 20 min for each participant. Play behaviors were assessed using procedures adapted from the literature [56]. Behaviors were recorded across two toy sets based on frequency and variety, then categorized as indiscriminate actions or discriminative actions. Finally, behaviors were classified as mastered, emerging, or absent according to predefined frequency and diversity criteria. Behaviors occurring under researcher or caregiver prompting were not included in the analysis.

### 2.3. Procedure

Ethical approval for the study was obtained from the Ethics Committee of Gazi University. Permissions for the use of all assessment tools, joint attention categories, and the Turkish Communicative Development Inventory (TIGE) were also obtained from the original developers. The informed consent forms for participation were distributed to the parents or legal guardians of all children and were signed prior to their inclusion in the study.

A pilot study was conducted to refine the observation procedures and coding system prior to the main data collection. The main study included toddlers aged between 16 and 36 months, comprising children with severe visual impairment and sighted peers. To enhance ecological validity, all observations were carried out in settings familiar to the children, primarily in their home environments. Data collection was conducted across multiple sessions. In the first session, children’s language development was assessed using the TIGE through structured interviews with primary caregivers. During this process, the researcher also interacted with the children to establish rapport and ensure their comfort.

In a subsequent session, joint attention and play behaviors were assessed. The researcher sat face-to-face with each child at their level and introduced the activities as a play interaction. Pre-determined materials were used to administer tasks corresponding to the joint attention categories. For children with severe visual impairment, materials were first introduced through tactile exploration prior to each task. Children’s joint attention behaviors were systematically recorded throughout the session.

Play skills were assessed through a semi-structured play interaction lasting approximately 20 min for each child. Two sets of toys were used, each presented for 10 min. During the play sessions, the researcher followed the child’s lead and avoided directing or intervening in the child’s play behaviors. For children with severe visual impairments, toys were introduced through tactile exploration before the play session, whereas no specific adaptations were made for sighted children.

### 2.4. Inter-Rater Reliability

Inter-rater reliability was calculated to assess the consistency between two independent coders. Approximately 30% of the video recordings were randomly selected and independently coded by a second trained researcher. The second coder was not involved in the data collection process and independently coded the selected video recordings. Reliability was computed using the formula: [Agreement/(Agreement + Disagreement)] × 100 [76]. Inter-rater reliability was 81.8% for joint attention behaviors and 86.3% for play behaviors.

### 2.5. Implementation Fidelity

Implementation fidelity was assessed to evaluate the extent to which the study procedures were implemented as planned. For this purpose, an implementation fidelity checklist was developed based on the study procedures. Approximately 30% of the video recordings were randomly selected and independently reviewed by a second trained researcher. The sample included five children with severe visual impairment and six sighted children. The second researcher coded whether each implementation step was observed or not observed. Implementation was calculated using the formula: [(Observed practitioner behavior/Planned practitioner behavior) × 100] [77]. Implementation fidelity was 90.9% for joint attention procedures and 93.6% for play behavior procedures.

### 2.6. Data Analysis

Data were analyzed using SPSS 20 software. Descriptive statistics were calculated for all variables. Due to the small sample size and non-normal distribution of the data, non-parametric analyses were conducted. Mann–Whitney U tests were used to compare the groups, and Spearman correlation analyses were performed to examine the relationships between joint attention and play variables.

## 3. Results

To examine differences in joint attention skills between toddlers with severe visual impairment and sighted peers, descriptive statistics are presented in Table 2, and the results of the Mann–Whitney U tests are provided in Table 3.

As presented in Table 3, the Mann–Whitney U test revealed significant differences between toddlers with severe visual impairment and sighted peers across all joint attention categories. Significant differences were observed in preliminary behaviors (U = 84.00, Z = −3.095, *p* = 0.002, r = 0.50), liberally construed joint attention behaviors (U = 65.50, Z = −3.311, *p* < 0.001, r = 0.54), conservatively construed joint attention behaviors (U = 23.00, Z = −4.623, *p* < 0.001, r = 0.76), initiating joint attention (U = 48.50, Z = −3.880, *p* < 0.001, r = 0.63), and responding to joint attention (U = 11.00, Z = −5.629, *p* < 0.001, r = 0.92).

The descriptive statistics presented in Table 2 indicate that toddlers with severe visual impairment obtained lower mean scores than sighted peers across all joint attention categories. In the preliminary behaviors category, toddlers with severe visual impairment (M = 2.29) scored lower than sighted peers (M = 2.85). In the liberally construed joint attention behaviors category, toddlers with severe visual impairment (M = 0.82) obtained lower scores than sighted children (M = 2.25). In the conservatively construed joint attention behaviors category, toddlers with severe visual impairment (M = 0.52) scored lower than sighted children (M = 2.85). Similarly, in the initiating joint attention category, toddlers with severe visual impairment (M = 0.70) obtained lower scores than sighted peers (M = 2.35). In the responding to joint attention category, toddlers with severe visual impairment (M = 0.47) also scored lower than sighted children (M = 2.95).

As presented in Table 4, significant differences were observed between toddlers with severe visual impairment and sighted peers in several play categories. Significant group differences were observed in indiscriminate actions (U = 44.50, Z = −3.826, *p* < 0.001, r = 0.63), discriminative actions (U = 17.00, Z = −4.758, *p* < 0.001, r = 0.78), takes apart combinations (U = 49.50, Z = −3.834, *p* < 0.001, r = 0.63), presentation combinations (U = 59.00, Z = −3.808, *p* < 0.001, r = 0.62), general combinations (U = 81.50, Z = −2.987, *p* = 0.003, r = 0.49), pretend self (U = 56.00, Z = −3.699, *p* < 0.001, r = 0.60), specific combinations (U = 81.00, Z = −3.261, *p* < 0.001, r = 0.53), child as agent (U = 29.00, Z = −4.621, *p* < 0.001, r = 0.76), and substitutions (U = 99.00, Z = −2.770, *p* = 0.006, r = 0.45).

The descriptive statistics presented in Table 5 indicate differences in mean scores between the groups across these categories. Toddlers with severe visual impairment obtained higher scores in indiscriminate actions (M = 138.11) compared with sighted peers (M = 47.35). In contrast, sighted children obtained higher mean scores in discriminative actions (M = 15.25 vs. M = 1.58), takes apart combinations (M = 7.80 vs. M = 1.35), presentation combinations (M = 5.45 vs. M = 0.23), and general combinations (M = 5.00 vs. M = 2.23). Sighted children also showed higher mean scores in pretend self (M = 3.65 vs. M = 0.58), specific combinations (M = 12.80 vs. M = 0.05), child as agent (M = 13.25 vs. M = 0.00), and substitutions (M = 2.50 vs. M = 0.05).

Spearman correlation analysis was conducted to examine the associations between joint attention skills and play skills in toddlers with severe visual impairment (Table 6).

Significant positive correlations were identified between preliminary behaviors and combinations (r = 0.596, *p* = 0.012) as well as pre-symbolic play (r = 0.598, *p* = 0.011). Liberally construed joint attention behaviors were positively associated with simple play (r = 0.512, *p* = 0.036), combinations (r = 0.592, *p* = 0.012), pre-symbolic play (r = 0.603, *p* = 0.010), and symbolic play (r = 0.588, *p* = 0.013). Conservatively construed joint attention behaviors were significantly associated with combinations (r = 0.535, *p* = 0.027) and pre-symbolic play (r = 0.785, *p* < 0.001). Initiating joint attention was positively related to combinations (r = 0.599, *p* = 0.011) and pre-symbolic play (r = 0.485, *p* = 0.049). Responding to joint attention was significantly associated with pre-symbolic play (r = 0.614, *p* = 0.009).

The associations between joint attention and play skills in sighted children were examined using Spearman correlation analysis (Table 7). The results indicated that liberally construed joint attention behaviors were positively associated with combinations (r = 0.458, *p* = 0.042) and symbolic play (r = 0.641, *p* = 0.002). In addition, conservatively construed joint attention behaviors were positively associated with symbolic play (r = 0.643, *p* = 0.002). Initiating joint attention was positively associated with pre-symbolic play (r = 0.524, *p* = 0.018). No other significant associations were observed between joint attention and play variables in the sighted group (*p* > 0.05).

## 4. Discussion

This study examined differences in joint attention and play skills between toddlers with severe visual impairments and sighted peers and investigated the associations between these developmental domains. The findings showed significant differences across all joint attention categories, with children with severe visual impairment obtaining lower scores than sighted peers. Differences were also observed in multiple dimensions of play development. In addition, joint attention and play skills were significantly associated in both groups, although the patterns of association varied.

Regarding joint attention, children with severe visual impairment scored lower in preliminary behaviors, liberally construed joint attention behaviors, conservatively construed joint attention behaviors, initiating joint attention, and responding to joint attention. These findings are consistent with previous research indicating delays or qualitative differences in joint attention development among children with visual impairments [33,34,35,78]. Limited access to visual cues such as gaze direction and pointing may constrain opportunities to coordinate attention with others [11,19,78]. As noted in earlier studies, children with severe visual impairment may rely on alternative strategies, such as auditory or tactile cues, which may not fully compensate for the absence of visual information in early joint attention development [38]. The large effect sizes observed across several joint attention categories suggest that these group differences are not only statistically significant but also developmentally meaningful.

Joint attention is considered a foundational developmental skill that emerges prior to symbolic language and supports early social-cognitive development [5,14]. Within early interactions, caregiver–child exchanges provide a critical context for the emergence of coordinated attention behaviors [16]. The present findings, showing lower performance in joint attention despite controlling for language developmental age, suggest that visual input plays a unique role beyond language abilities. This is in line with research emphasizing that vision facilitates the detection of others’ attentional focus and supports shared engagement [20,21].

Significant differences were also found in play development. Children with severe visual impairment demonstrated higher levels of indiscriminate actions, whereas sighted peers showed higher scores in more advanced play categories. These findings are consistent with previous studies reporting that children with visual impairments tend to engage less frequently in symbolic and socially interactive forms of play [48,50,53,54,79,80]. Limited access to visual information may reduce opportunities for observational learning and imitation, which are important for the development of symbolic play [26,42]. Additionally, these children often rely more on tactile and auditory exploration, which may support early play behaviors but may not fully facilitate the transition to more complex representational play forms.

The associations between joint attention and play skills were also examined. In children with severe visual impairment, joint attention categories were positively associated with pre-symbolic play, whereas in sighted children, joint attention was more strongly associated with higher-level play behaviors, including symbolic play. These findings are consistent with developmental models suggesting that joint attention supports the emergence of symbolic and representational play [56,57]. Previous research has also demonstrated strong links between joint attention, imitation, and symbolic functioning [57,58]. The present results extend this literature by showing that, in children with severe visual impairment, these associations may be more evident at earlier developmental levels, reflecting differences in developmental trajectories. By comparing children matched on language developmental age, the present findings suggest that visual experience may play an important role in the development of joint attention and play skills during early childhood. Although the groups were similar in language developmental age, children with severe visual impairment demonstrated lower performance in multiple joint attention and play skills, highlighting the potential contribution of vision to early social-cognitive development.

Several limitations should be noted. The sample included children aged 16–36 months, which limits generalization beyond this age range. The sample size was relatively small due to limited access to eligible participants. In addition, etiological differences underlying severe visual impairment were not examined separately in the present study. In addition, joint attention behaviors were evaluated based on presence rather than frequency. Future research may include longitudinal designs, larger samples, comparisons with children with low vision or additional disabilities, and naturalistic observation methods. Intervention studies examining programs targeting joint attention, play, and communication skills are also recommended. These findings highlight the importance of early intervention programs targeting joint attention and play skills in children with severe visual impairment.

## 5. Conclusions

This study examined differences in joint attention and play skills between toddlers with severe visual impairment and sighted peers and investigated the associations between these developmental domains. The findings demonstrated that children with severe visual impairment obtained consistently lower scores across all joint attention categories, including Preliminary Behaviors, Liberally Construed Joint Attention Behaviors, Conservatively Construed Joint Attention Behaviors, Initiating Joint Attention, and Responding to Joint Attention. These results suggest a differentiated performance profile in early joint attention skills within the examined age range.

Group differences were also evident in play development. Children with severe visual impairment showed higher levels of indiscriminate actions, whereas sighted children demonstrated higher performance in more advanced play categories. These findings suggest differences in the developmental progression of play behaviors between the groups.

The analysis of associations between joint attention and play skills indicated that these domains are related in both groups. In children with severe visual impairment, significant associations were primarily observed between joint attention skills and lower-level play behaviors, whereas in sighted children, joint attention was associated with higher-level play behaviors. These results suggest that the relationship between joint attention and play differs in structure depending on visual status.

Overall, the findings provide a comprehensive characterization of joint attention and play development in toddlers with and without severe visual impairment. The results highlight differences not only within individual developmental domains but also in the patterns of association between them. These findings have important implications for early assessment and intervention, emphasizing the need to support joint attention and play skills in children with severe visual impairment. Early intervention practices may particularly support joint attention, reciprocal interaction, tactile exploration, and play-based social engagement in children with severe visual impairment through structured interaction opportunities [81].

## Figures and Tables

**Table 1:** Demographic characteristics of the participants.

Group	n	Age (Months) M	SD	Min–Max	Girls n (%)	Boys n (%)
Severe visual impairment	17	27.70	1.47	19–36	10 (59%)	7 (41%)
Sighted	20	25.45	1.14	17–33	11 (55%)	9 (45%)

**Table 2:** Descriptive statistics of joint attention skills in participant children.

Joint Attention Category	Group	N	Mean (X)	SD	Variance	Min	Max	Skewness	Kurtosis
Preliminary Behaviors	SVI	17	2.29	0.82	0.58	1.00	3.00	−0.10	−0.32
	S	20	2.85	1.18	1.40	0.00	3.00	−2.12	2.77
Liberally Construed Joint Attention Behaviors	SVI	17	0.82	1.00	1.00	1.00	3.00	1.14	−0.24
	S	20	2.25	0.81	0.66	0.00	3.00	0.29	−1.10
Conservatively Construed Joint Attention Behaviors	SVI	17	0.52	0.91	0.84	0.00	4.00	1.98	0.87
	S	20	2.85	1.03	1.08	0.00	4.00	0.29	0.78
Initiating Joint Attention	SVI	17	0.70	0.34	0.12	0.00	3.00	1.21	0.87
	S	20	2.35	0.13	0.02	0.00	3.00	−1.42	0.78
Responding to Joint Attention	SVI	17	0.47	0.71	0.51	0.00	2.00	1.26	0.39
	S	20	2.95	0.22	0.05	2.00	5.00	−4.47	5.00

**Table 3:** Mann–Whitney U Test results for joint attention skills.

Joint Attention Category	Group	N	SD	Mean Rank	Sum of Ranks	Z	M	U	*p*
Preliminary Behaviors	SVI	17	0.58	13.94	237.00	−3.095	3.00	84.00	0.002 **
	S	20	0.36	23.30	466.00				
Liberally Construed Joint Attention Behaviors	SVI	17	1.18	12.85	218.50	−3.311	1.00	65.50	0.001 **
	S	20	1.16	24.23	484.50				
Conservatively Construed Joint Attention Behaviors	SVI	17	1.00	10.35	176.00	−4.623	2.00	23.00	0.000 ***
	S	20	0.81	26.35	527.00				
Initiating Joint Attention	SVI	17	0.91	11.85	201.50	−3.880	2.00	48.50	0.000 ***
	S	20	1.03	25.08	501.50				
Responding to Joint Attention	SVI	17	0.71	9.06	154.00	−5.629	3.00	11.00	0.000 ***
	S	20	0.22	27.45	549.00				

* *p* < 0.05, ** *p* < 0.01, *** *p* < 0.001.

**Table 4:** Mann–Whitney U Test results for play skills.

Play Categories	Group	N	SD	Mean Rank	Rank Sum	Z	U	*p*
Indiscriminate actions	SVI	17	78.16	26.38	448.50	−3.826	44.50	<0.001 ***
	S	20	30.12	12.73	254.00			
Discriminative actions	SVI	17	4.00	10.00	170.00	−4.758	17.00	<0.001 ***
	S	20	8.21	26.65	533.00			
Takes apart combinations	SVI	17	4.34	11.91	202.50	−3.834	49.50	<0.001 ***
	S	20	5.03	25.03	500.50			
Presentation combinations	SVI	17	0.97	12.47	212.00	−3.808	59.00	<0.001 ***
	S	20	8.14	24.55	491.00			
General combinations	SVI	17	6.55	13.79	234.50	−2.987	81.50	0.003 **
	S	20	7.05	23.43	468.50			
Pretend self	SVI	17	1.93	12.29	209.00	−3.699	56.00	<0.001 ***
	S	20	2.92	24.70	494.00			
Specific combinations	SVI	17	0.24	13.76	234.00	−3.261	81.00	0.001 **
	S	20	17.31	23.45	469.00			
Child as agent	SVI	17	0.72	10.71	182.00	−4.621	29.00	<0.001 ***
	S	20	12.24	26.05	521.00			
Specific conventional combinations	SVI	17	0.97	19.59	333.00	−1.085	160.00	0.278
	S	20	0.00	18.50	370.00			
Single-scheme sequences	SVI	17	0.00	19.00	323.00	0.000	170.00	1.000
	S	20	0.00	19.00	380.00			
Substitutions	SVI	17	0.24	14.82	252.00	−2.770	99.00	0.006 **
	S	20	3.05	22.55	451.00			
Doll as agent	SVI	17	0.00	18.50	314.50	−0.922	161.50	0.357
	S	20	0.89	19.43	388.50			
Multischeme sequences	SVI	17	0.00	19.00	323.00	0.000	170.00	1.000
	S	20	0.00	19.00	380.00			
Sociodramatic play	SVI	17	0.00	19.00	323.00	0.000	170.00	1.000
	S	20	0.00	19.00	380.00			
Thematic fantasy play	SVI	17	0.00	19.00	323.00	0.000	170.00	1.000
	S	20	0.00	19.00	380.00			

* *p* < 0.05, ** *p* < 0.01, *** *p* < 0.001.

**Table 5:** Descriptive statistics for play skills of participating children.

Play Categories	Group	N	M	SD	Variance	Min	Max	Skewness	Kurtosis
Indiscriminate actions	SVI	17	138.11	78.16	6109.23	19.00	283.00	0.467	−0.743
	S	20	47.35	30.12	907.50	10.00	131.00	1.01	1.50
Discriminative actions	SVI	17	1.58	4.00	16.00	0.00	14.00	2.71	6.58
	S	20	15.25	8.21	67.56	5.00	34.00	1.50	1.24
Takes apart combinations	SVI	17	1.35	4.34	18.86	0.00	18.00	3.96	16.00
	S	20	7.80	5.03	25.32	0.00	16.00	−0.161	−1.05
Presentation combinations	SVI	17	0.23	0.97	0.94	0.00	4.00	4.12	17.00
	S	20	5.45	8.14	66.26	0.00	36.00	3.00	10.93
General combinations	SVI	17	2.23	6.55	42.94	0.00	24.00	2.96	8.39
	S	20	5.00	7.05	49.78	0.00	27.00	2.10	4.44
Pretend self	SVI	17	0.58	1.93	3.75	0.00	8.00	3.93	15.82
	S	20	3.65	2.92	8.55	0.00	10.00	0.539	−0.156
Specific combinations	SVI	17	0.05	0.24	0.05	0.00	1.00	4.12	17.00
	S	20	12.80	17.31	299.74	0.00	58.00	1.46	1.44
Child as agent	SVI	17	0.00	0.00	0.00	0.00	0.00	4.12	17.00
	S	20	13.25	12.24	149.88	0.00	39.00	0.801	−0.551
Specific conventional combinations	SVI	17	0.23	0.97	0.94	0.00	4.00	4.12	17.00
	S	20	0.00	0.00	0.00	0.00	0.00	0.00	0.00
Single-scheme sequences	SVI	17	0.00	0.00	0.00	0.00	0.00	0.00	0.00
	S	20	0.00	0.00	0.00	0.00	0.00	0.00	0.00
Substitutions	SVI	17	0.05	0.24	0.05	0.00	1.00	4.12	17.00
	S	20	2.50	3.05	9.31	0.00	7.00	0.55	−1.62
Doll as agent	SVI	17	0.00	0.00	0.00	0.00	0.00	0.00	0.00
	S	20	0.20	0.89	0.80	0.00	4.00	4.47	20.00
Multischeme sequences	SVI	17	0.00	0.00	0.00	0.00	0.00	0.00	0.00
	S	20	0.00	0.00	0.00	0.00	0.00	0.00	0.00
Sociodramatic play	SVI	17	0.00	0.00	0.00	0.00	0.00	0.00	0.00
	S	20	0.00	0.00	0.00	0.00	0.00	0.00	0.00
Thematic fantasy play	SVI	17	0.00	0.00	0.00	0.00	0.00	0.00	0.00
	S	20	0.00	0.00	0.00	0.00	0.00	0.00	0.00

**Table 6:** Correlations between joint attention skills and play skills in toddlers with severe visual impairment.

Joint Attention Skills	Statistic	Simple Play	Combinations	Pre-Symbolic Play	Symbolic
Preliminary Behaviors	r	0.190	0.596	0.598	0.472
	*p*	0.465	0.012 *	0.011 *	0.056
Liberally Construed Joint Attention Behaviors	r	0.512	0.592	0.603	0.588
	*p*	0.036 *	0.012 *	0.010 *	0.013 *
Conservatively Construed Joint Attention Behaviors	r	0.126	0.535	0.785	0.116
	*p*	0.630	0.027 *	<0.001 *	0.657
Initiating Joint Attention	r	0.346	0.599	0.485	0.389
	*p*	0.174	0.011 *	0.049 *	0.122
Responding to Joint Attention	r	0.250	0.373	0.614	0.066
	*p*	0.334	0.141	0.009 *	0.801

* *p* < 0.05.

**Table 7:** Correlations between joint attention skills and play skills in sighted children.

Joint Attention Skills	Statistic	Simple Play	Combinations	Pre-Symbolic Play	Symbolic
Preliminary Behaviors	r	0.216	0.228	0.279	−0.223
	*p*	0.316	0.334	0.234	0.346
Liberally Construed Joint Attention Behaviors	r	−0.168	0.458	0.252	0.641
	*p*	0.479	0.042 *	0.284	0.002 *
Conservatively Construed Joint Attention Behaviors	r	−0.078	0.306	0.116	0.643
	*p*	0.745	0.190	0.625	0.002 *
Initiating Joint Attention	r	−0.300	−0.186	0.524	0.098
	*p*	0.199	0.433	0.018 *	0.682
Responding to Joint Attention	r	−0.096	−0.022	0.168	0.343
	*p*	0.686	0.927	0.478	0.139

* *p* < 0.05.

## Data Availability

The data supporting the findings of this study are available upon request from the corresponding author. They are not publicly available due to privacy or ethical restrictions.
